# Psychosocial issues of women with type 1 diabetes transitioning to motherhood: a structured literature review

**DOI:** 10.1186/1471-2393-13-218

**Published:** 2013-11-23

**Authors:** Bodil Rasmussen, Christel Hendrieckx, Brydie Clarke, Mari Botti, Trisha Dunning, Alicia Jenkins, Jane Speight

**Affiliations:** 1 School of Nursing and Midwifery, Deakin University, 221 Burwood Highway, Burwood, Vic 3125, Australia; 2 The Australian Centre for Behavioural Research in Diabetes, Diabetes Australia – Vic, 570 Elizabeth Street, Melbourne, VIC 3000, Australia; 3 Centre for Mental Health and Well-being Research, School of Psychology, Deakin University, 221 Burwood Highway, Burwood, Vic 3125, Australia; 4 Deakin University, Waterfront, PO Box 281, Geelong, Vic 3220, Australia; 5 Department of Medicine, St Vincent’s Hospital, 4th Floor Clinical, Science Building, Melbourne University, 29 Regent Street, Fitzroy, Vic 3065, Australia; 6 AHP Research, 16 Walden Way, Hornchurch, UK; 7 Epworth Health Care, Centre for Clinical Research Nursing, Epworth, Australia

**Keywords:** Type 1 diabetes mellitus, Woman, Literature review, Pregnancy, Life transition, Motherhood, Social support, Well-being

## Abstract

**Background:**

Life transitions often involve complex decisions, challenges and changes that affect diabetes management. Transition to motherhood is a major life event accompanied by increased risk that the pregnancy will lead to or accelerate existing diabetes-related complications, as well as risk of adverse pregnancy outcomes, all of which inevitably increase anxiety. The frequency of hyperglycaemia and hypoglycaemia often increases during pregnancy, which causes concern for the health and physical well-being of the mother and unborn child. This review aimed to examine the experiences of women with T1DM focusing on the pregnancy and postnatal phases of their transition to motherhood.

**Methods:**

The structured literature review comprised a comprehensive search strategy identifying primary studies published in English between 1990–2012. Standard literature databases were searched along with the contents of diabetes-specific journals. Reference lists of included studies were checked. Search terms included: ‘diabetes’, ‘type 1’, ‘pregnancy’, ‘motherhood’, ‘transition’, ‘social support’, ‘quality of life’ and ‘psychological well-being’.

**Result:**

Of 112 abstracts returned, 62 articles were reviewed in full-text, and 16 met the inclusion criteria. There was a high level of diversity among these studies but three common key themes were identified. They related to physical (maternal and fetal) well-being, psychological well-being and social environment. The results were synthesized narratively.

**Conclusion:**

Women with type 1 diabetes experience a variety of psychosocial issues in their transition to motherhood: increased levels of anxiety, diabetes-related distress, guilt, a sense of disconnectedness from health professionals, and a focus on medicalisation of pregnancy rather than the positive transition to motherhood. A trusting relationship with health professionals, sharing experiences with other women with diabetes, active social support, shared decision and responsibilities for diabetes management assisted the women to make a positive transition. Health professionals can promote a positive transition to motherhood by proactively supporting women with T1DM in informed decision-making, by facilitating communication within the healthcare team and co-ordinating care for women with type 1 diabetes transitioning to motherhood.

## Background

Diabetes is one of Australia’s leading health priority areas with a high health and cost burden. Approximately 130,000 Australians (0.4% of the Australian population) have type 1 diabetes (T1DM) [1]. Around 47% of these are less than 45 years old and just under 47% are female [2]. Thus, the prevalence of T1DM among women of child-bearing age (15–49 years) accounts for nearly 23,000 women with T1DM in that age group [2]. The combination of T1DM and pregnancy can cause specific complications for mother and baby [2]. Pre-existing diabetes in pregnancy affects less than 1% of all pregnancies in Australia but serious short- and long-term complications increase likelihood of hospitalisation and death, impairment of quality of life (QoL) and well-being [2]. Pre-existing diabetes-related complications can be accelerated by pregnancy [3] and women with T1DM are at high risk of pregnancy-related complications [4-6]. Babies of mothers with T1DM have higher rates of still birth, pre-term birth, high birth weight, admission to special care and neonatal intensive care units, and longer stay in hospital compared to babies of mothers with other types of diabetes or mothers without diabetes [2].

Transition to motherhood is a major life-changing event and a common concept in developmental, stress, and adaptation theories [7,8]. Transitions accommodate both the continuities and discontinuities in life processes and are invariably related to change and development [9]. Transitions have many characteristics, including how they are experienced, their developmental and growth value, and their function linking people to their social context [10]. Transitions and changes in health status can be difficult and oppressive but they also represent opportunities to enhance quality of life [3,7,8,11-14]. New experiences can help people change from a maladaptive life trajectory to a more adaptive trajectory. Metaphorically speaking, life is a path and for most people the path is not straight.

Life transitions are usually associated with significant change, complex decisions and increased stress, which can affect problem-solving and coping abilities [14,15]. The added stress is particularly concerning during transition to motherhood because managing blood glucose levels (BGLs) is intense and difficult for women with T1DM and can result in hyper- or hypoglycaemia-related emergencies [14,16]. The frequency of hypoglycaemia often increases during pregnancy, which causes concern for the health and physical well-being of the mother and unborn child [17-19]. In women with T1DM, severe hypoglycaemia occurs three to five times more frequently in early pregnancy than in the period prior to pregnancy, whereas in the third trimester the incidence of severe hypoglycaemia is lower than in the year preceding pregnancy [16]. Risk factors for severe hypoglycaemia during pregnancy include a history of severe hypoglycaemia in the year preceding pregnancy, impaired awareness of hypoglycaemia, long duration of diabetes, low HbA1c in early pregnancy, fluctuating plasma glucose values and excessive use of supplementary insulin injections between meals [5,16].

Pregnant women with T1DM are often more anxious about their pregnancies and perceive their risks for adverse pregnancy outcomes as high compared with women without diabetes [19-21]. Transition to motherhood is, therefore, particularly challenging for women with T1DM because preparing for and going through pregnancy requires rigorous diabetes management and meticulous planning of everyday activities [22].

While numerous studies focus on the physical and medical aspects of pregnancy in women with T1DM, fewer are concerned with their psychosocial experiences and needs. Three studies have emerged in recent years [19,22,23], focusing on psychosocial aspects of transition to motherhood for women with T1DM. However, only one of the studies addressed the transition period comprising both the pregnancy and postnatal phases into motherhood with the focus on health services [19]. Thus, the aim of the present structured literature review was to examine the experiences of women with T1DM focusing on the pregnancy and postnatal phases of their transition to motherhood.

## Methods

### Search methods

A comprehensive search strategy was used and the following search terms applied: (‘diabetes’ OR ‘type 1’) AND (‘pregnancy’ OR ‘motherhood’ OR ‘transition’) AND (‘social support’ OR ‘quality of life’ OR ‘psychological well-being’). The following databases were searched to identify papers published between 1990–2011 in English: PUBMED, CINAHL, EMBASE, Web of Science, EBSCO Host databases, Expanded Academic, ASAP, Rand, Science Direct (Elsevier), Cochrane Library (Wiley Interscience), Google Scholar, Dissertation and Theses. Manual searches were undertaken of the contents pages of diabetes-specific journals (e.g. *Diabetes Care*, *The Diabetes Educator*, *Diabetic Medicine*, *Diabetes Research and Clinical Practice*, *Diabetes Spectrum, Journal of Diabetes Nursing*) and the reference lists of studies meeting the inclusion criteria. Studies were selected for inclusion in the review if they met the following criteria:

•Empirical (quantitative or qualitative) studies concerning the experiences of women with T1DM:

–during pregnancy, or

–during the postnatal period.

Studies were excluded if they:

•did not specifically report on diabetes

•concerned gestational diabetes only or type 2 diabetes only

•concerned T1DM but did not include relevant data about the transition to motherhood (during pregnancy or the postnatal period) or focused only on planning pregnancy

•did not report on the psychosocial aspects of transitions and/or only reported biomedical outcomes

•focused on women of Aboriginal or Torres Strait Islander (ATSI) origin, as cultural, social and psychological issues differ in this group and are beyond the remit of this review

•were reviews, commentaries, editorials or letters.

### Data abstraction

One investigator screened abstracts and selected studies for full-text review and a second reviewer cross-checked for agreement. Each full-text article was reviewed at least twice by two reviewers (BR and BC) and later by a third reviewer (CH) and differences were resolved through discussion. A table summarising selected studies was reviewed by all authors, comprising researchers, consumer representatives and clinicians. The following data were abstracted for each study: Author(s), year of publication; country; title; study design; aims, methods and outcome measures; sample size, age and ethnicity of participants; and key findings.

### Quality appraisal

All studies were reviewed for methodological rigour by more than one investigator. Due to diverse research designs, it was not appropriate to conduct a meta-analysis, or to apply a single critical appraisal tool to all studies. Thus, a structured review process was informed by guidelines for conducting critical appraisal of quantitative and qualitative research [24] involving a sequential five step process:

1. Comprehension: skimming and reading through articles to grasp key ideas and content.

2. Comparison: reading each selected article to understand the research problem, aim, design, sample size, data-collection procedure and key findings.

3. Analysis: examining the logical links connecting with the review’s aim and inclusion/exclusion criteria.

4. Evaluation: determining the meaning, significance, and validity by examining links between the study process, findings, and previous studies.

5. Conceptual clustering: synthesizing the studies’ findings in the transitional framework of pre-pregnancy, during pregnancy and the postnatal phase.

**Figure 1:**
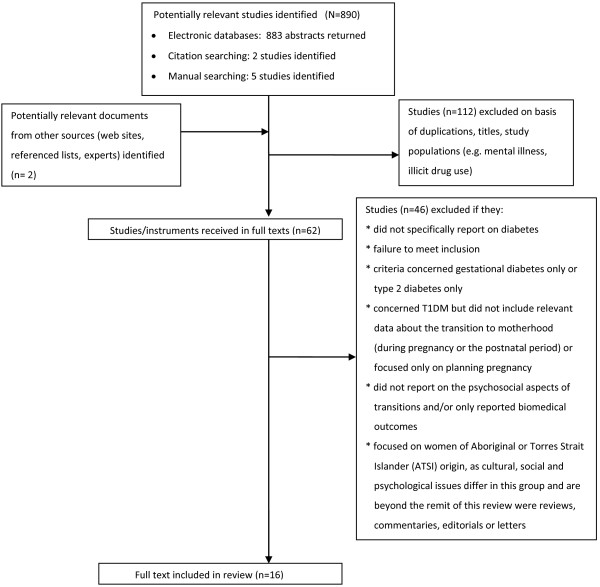
Process of selecting studies for the review.

There was some congruence among the papers and a number of key findings emerged within transitional phases. A flowchart of the process of selecting studies for the review is provided in Figure 1.

Ethics approval was granted by Deakin University Human Ethics Committee (HEAG-H 126_2010).

## Results

### Search outcome and appraisal of studies

A total of 890 abstracts were identified as potential for inclusion of the review. After the abstract duplicates removed and checked against inclusion criteria, 112 abstracts were identified as relevant but not matching all inclusion criteria, 62 articles were selected for full-text review and 16 met the all inclusion criteria. An additional study was identified during the peer review process. An overview of the 16 studies is provided in Table 1, while a detailed summary with methodological details of each study (design, sample, and key findings) is presented in Additional file 1.

**Table 1:** Summary of sample size (T1DM sample only) and transition phase investigated, by study design (qualitative vs quantitative) included reviewed articles

**Authors & year**	**Country**	**Sample (N) T1DM only**	**Transition phase**^**1**^				
			**Pregnancy (trimester)**			**Immediately post-partum (up to 4 weeks)**	**Post-natal (>4 weeks)**
			**1**	**2**	**3**		
Qualitative studies							
Berg M & Honkasalo ML [25]	Sweden	14	X	X	X	X	
Berg M [26]	Sweden	18	X	X	X	X	
Berg M & Sparud-Lundin C [27]	Sweden	23					X
King R & Wellard S [28]	Australia	7					X
Lavender T et al. [23]	UK	22					
Sparud-Lundin C & Berg M [22]	Sweden	23					X
Stenhouse et al. [19]	UK	8	X	X	X		
Quantitative studies:							
Moore ML et al. [33]	USA	73		X	X		
Spirito A et al.^2^[35]	USA	NR		X	X		
Ruggiero L et al. [36]	USA	33		X	X		
York R et al. [37]	USA	NR				X	X
Langer N & Langer O [31,38]	USA	NR			X		
Levy-Shiff R et al. [32]	Israel	NR		X			
Illias J et al. [30]	Greece	33		X	X		
Dalfra MG et al. [29]	Italy	30					X
Sparud-Lundin C et al. [34]	Sweden	105					X

^1^Qualitative studies were often conducted at the post-natal phase but, in some cases, required the women to reflect on experiences during and pre-pregnancy.

^2^Included quantitative assessment using Profile of Mood States (POMS) questionnaire.

*NR*: not reported.

Seven were qualitative studies [19,22,23,25-28] while nine used quantitative methods [29-37]. The majority of studies were conducted in Sweden [22,25-27,34] and the USA [31,33,35-38], and the remaining studies were conducted in Australia [28], Greece [30], Israel [32], Italy [29], and the UK [19,23]. The focus of nine studies was the pregnancy itself [25,26,30-33,35,36,38], while three focused on the immediate post-partum phase [25,26,37] and six focused on the postnatal phase [22,27-29,34,37] as illustrated in Table 1.

None of the studies included women pre-pregnancy, though some qualitative studies asked women at a later phase to reflect back on that period. Six studies [29-32,37,38] included women with gestational diabetes as well as those with T1DM but our current review focused only on data relating to women with T1DM. Samples of women with T1DM ranged N = 7-23 in the qualitative studies and N = 30-105 in the quantitative studies. In five studies [31,32,35,37,38], it was not possible to determine the exact number of women with T1DM included.

### Key findings

The findings relate broadly to three main themes: physical well-being (maternal and fetal), psychological well-being, and social environment. The findings are presented within each theme according to the two transitional phases: during pregnancy and the postnatal period (including the limited information available about women’s experience during childbirth).

### Physical well-being (maternal and fetal well-being)

The qualitative studies indicated that diabetes deprived women of the normal experiences associated with pregnancy and being a ‘mother-to-be’. Some women felt their baby’s health depended on their commitment to manage their diabetes [25]. The women’s perception that they and their baby were at risk because of diabetes was escalated by the primarily medical care they received.

#### During pregnancy

During pregnancy, one of the women’s main concerns was the sense of losing control of their body and their diabetes [23,31]. Women felt they could no longer rely on their bodily cues, especially recognising hypoglycaemic symptoms [28]. Not recognising or trusting their bodily cues made it difficult to manage fluctuating BGLs [25,28]. To compensate for unpredictable BG fluctuations, women monitored their BGLs more frequently, which disrupted their daily routines and their sleep [23,25,28].

Daily life was dominated by trying to normalise BGLs [28,36] primarily for the sake of the unborn baby but also to reduce the women’s own complication risk [25]. Managing their diabetes and the welfare of their unborn child required a more routine lifestyle (e.g. regular eating, sleeping and physical activities), changing workplaces or taking sick leave, which was new for some women [25].

The women’s increased awareness of diabetes led to extensive behavioural changes because of modified insulin management and increased number of health service visits [26,28,36]. Despite the many challenges, most women felt strongly motivated to manage their diabetes [28,35]. Their hope and desire for a healthy child was a strong motivator and made the extra work worth the effort [25]. Well-being was enhanced when women “mastered” the specific challenges during their pregnancy instead of being “enslaved” by them [26].

Pregnant women with T1DM and those with GDM reported better physical health than pregnant women without diabetes [29]. The increased attention on the women’s health because of their diabetes might partly explain the finding. Women with T1DM scored better than those without diabetes for physical functioning, bodily pain and general health perception. However, it should be noted that the mean physical health scores of the women without diabetes were relatively low and therefore inconclusive.

#### Postnatal

Maintaining glycaemia stability continued to be a challenge in the postnatal phase. Women felt a strong need to monitor and control their BGLs to maintain their capacity to care for their child [34]. The women feared unexpected hypoglycaemia would compromise their ability to care for their child and, therefore, took measures to prevent hypoglycaemia. Fluctuating BGLs became particularly difficult while breast-feeding and the women, therefore, prioritised control of diabetes (e.g. by eating prior to breast-feeding) so they could continue to breast-feeding without risk of hypoglycaemia [34].

In summary, one of the major physical challenges for pregnant women with diabetes throughout the whole transition process was managing BGLs.

### Psychological well-being

Women with diabetes were at increased risk of becoming distressed during pregnancy because of the associated uncertainty, which in turn had a significant effect on psychological well-being. Women with pre-existing diabetes reported that pregnancy placed a greater strain on them compared to pregnant women without diabetes [28,34]. Increased ambivalence or doubts, coupled with fear of losing the unborn child, can interfere with the normal adjustment to pregnancy [32] and can trigger a sense of disempowerment, high levels of worrying, depression, guilt and fear of being a ‘burden’ to others [25,27,35].

#### During pregnancy

Pregnant women with T1DM experienced greater anxiety and depressive moods [35,38], were more distressed [33] and reported lower mental health [29] compared to pregnant women without pre-existing diabetes. They also reported more intense pregnancy-related negative feelings and fewer positive emotions than pregnant women without diabetes. However, these higher levels of anxiety and depression were not clinically relevant [32].

It was not clear whether the emotional profiles of women with pre-existing diabetes and women with gestational diabetes differed. During pregnancy, women with T1DM reported greater anxiety and more depressive and hostile moods compared to women with gestational diabetes [30,38]. In contrast, others found that, during pregnancy, women with gestational diabetes had slightly higher anxiety and hostility scores than women with pre-gestational diabetes, but did not have higher depression scores. Overall, women in both groups did not report clinically relevant impaired psychological well-being [37]. However, there was no comparator with pregnant women without diabetes, and the number of women with T1DM was very small.

#### Postnatal

Depressive symptoms and worsening mental health status were significant changes for women with T1DM [29]. After delivery, health status worsened and these women may have had greater difficulty recovering, due to fluctuating BGLs [29]. It is also possible that being aware of their diabetes and the continual BG monitoring could induce worry about their ability to cope with motherhood and take adequate care of their child. Some women might also worry about attachment and bonding issues [29].

In summary, psychological well-being in women with T1DM during their transition to motherhood was affected by anxiety, distress and depression. Although strong emotions in the transition to motherhood are well documented among women without diabetes, the current review indicates emotional distress escalates in women with T1DM in the postnatal period. Therefore, the women’s social environment and received support are crucial components during the whole transition process.

### Social environment

Social support is important for all women transitioning to motherhood. The individualised diabetes management regimen during this period influences the woman’s social support needs. The review findings suggest that the women’s need for social support, in particular from partners and health professionals, were influenced by their access to relevant, accurate information and to appropriate services.

#### During pregnancy

Health professionals play an important role in helping women achieve optimal pregnancy outcomes, including helping them develop the confidence to manage their diabetes during their pregnancy and post-natally [19,23,27,29]. As a result of frequent hypoglycaemia and unstable BGLs, the women relied, especially on their partners and close friends to help manage daily activities. Some women were afraid to sleep alone because of their fear for hypoglycaemia [25].

Women with T1DM also found themselves being a ‘messenger’ between various health professionals, which lead to distrust and uncertainty concerning some health professionals’ knowledge and competence. Consequently, the women felt they needed to become their own diabetes expert [27]. Women became frustrated when health professionals did not value this expertise. Pregnant women with T1DM viewed themselves to be experts in their own diabetes but the changes during pregnancy made them rely more on partners and family members, particularly their mothers, who they also felt had expertise in their diabetes management [19]. Pregnant women with diabetes with T1DM reported that any demonstration of their or their significant others’ expertise, particularly during labour, was met with resistance which upheld the expert/patient dichotomy.

During acute care episodes, such as labour, this led to high stress levels for the women and a feeling of distrust towards the health professionals [19,27]. Moreover, women hesitated to raise their pregnancy-related concerns in fear of being judged, as they perceived their consultation with health professionals not as a dialog or open exchange of ideas but rather a session in which health professionals, advised, judged and exerted control. In addition, the focus in the interaction with health professionals was on BGLs rather than the whole person and the situation of being pregnant [19], which exacerbated women’s sense of mistrust of and disconnectedness from health professionals. The pregnancy period was ‘frustrating’ because women felt they lost control to a care system, which disrupted their everyday lives and forced them to surrender their control to health professionals and influential others [23].

Sometimes, the information health professionals provided increased these concerns [28,29,34] because it was discouraging, inadequate or inaccurate. Some women did not receive any information [28,34]. Women living in rural communities experienced additional difficulties accessing knowledgeable health professionals and services; consequently, travel to available services represented a cost and time burden and exacerbated stress [28]. However, women who were supported and acknowledged for their efforts by their medical team were highly satisfied [28] and reported fewer negative emotions [32].

In spite of evidence that stress and social support affect health behaviours and pregnancy outcomes, few studies examined the effect of social support during pregnancies in T1DM. Women were more likely to follow a recommended diet plan when social support was provided and stress was perceived as manageable [36].

Women shared experiences and sought support from other pregnant women with diabetes. Some felt lonely during their pregnancy [27] and used the internet to seek diabetes-related information and communicate with other women [34]. The informal, emotional and appraisal support from women in similar situations fostered a sense of belonging [22,27]. The web-based support for child-bearing women with T1DM was very important provided it contained reliable information, improved access to health professionals, offered interactive support and social networking during pregnancy and after they gave birth [34].

#### Postnatal

Women’s need for support changed in the postnatal phase; they felt abandoned by or disconnected from health professionals [22]. A trusting relationship with a health professional as well as sharing their experiences with other women with T1DM were essential to the women’s confidence in managing their BGLs [27].

Specific support early postpartum and after discharge from maternity care was of the utmost importance. Support from a partner was essential to enable women to manage daily life with a newborn baby, their diabetes and breast-feeding but the women also needed to maintain a sense of control [22]. Women felt a duality between their need for support from health professionals and partners, and their need to take responsibility for their BGLs during and immediately after the birth. The authors emphasised that support needed to be negotiated and that responsibility for glucose management needed to be clarified between the woman, her health professional and partner.

In summary, social environment plays an integral role in women’s perception of stress, their sense of control over their diabetes, and their transition to motherhood in general. Social support is optimal when it meets women’s needs and recognises their knowledge and capabilities, helps to build trusting relationships with health professionals and involves partners.

## Discussion

We identified seven qualitative and nine quantitative studies examining the experiences of women with T1DM as they transition to motherhood. While most studies included modest numbers of women with T1DM, this review reveals the needs of women with T1DM as they move through pregnancy and beyond, to life with a newborn baby.

Earlier research into the transition to motherhood of women with T1DM established that women’s decisions or choice of strategies for managing the transition is based on their perceptions of the impact life changes have on their psychological well-being [6,14,39]. This review has found that women with T1DM experience a variety of psychosocial issues in their transition to motherhood, including increased anxiety, diabetes-related distress, guilt, and sense of disconnectedness from health professionals.

### Transitioning into motherhood: a matter of “negotiated” team work

The transition to motherhood is an important opportunity for health professionals and women with T1DM to collaborate in achieving good health outcomes for both mother and child. Despite many challenges during transition to motherhood, women felt strongly motivated to manage their diabetes because having a baby made their self-care effort purposeful [25,26,28]. The women’s sense of control over their diabetes was directly linked to their physical, psychological and social well-being.

Although health professionals have the medical knowledge and skills to minimise adverse outcomes of pregnancy, the main responsibility belongs to the woman with T1DM. The responsibility of managing diabetes has been identified elsewhere as ‘being in the grip of BGLs’ [14]. The women felt the grip of BGLs to be more or less tight depending on the environmental and social context they found themselves in at the time of the transition. When women had difficulty controlling their BGLs, e.g. experiencing hypoglycaemia, they experienced a complete loss of control, and they sought assistance from health professionals and health services more frequently. The responsiveness of health services was a key component of being in the grip of BGLs, because it was even tighter when the women felt the health services were not responsive to their particular needs [14].

The transition to motherhood can be described as an opportunity for pregnant women with T1DM to re-evaluate their lives, including their life goals, priorities, and preferences [6,17]. Health professionals do not always recognise the changing needs of women through the different stages of pregnancy; potentially, creating mistrust of health professionals and anxiety in the women [18,22,31,37]. While the importance of vigilance in relation to monitoring of BGLs is well recognised by women with T1DM, they are frustrated by the absence of focus on their individual pregnancy experiences. Women were keen to ask questions relating to pregnancy-specific topics, such as labour, but they felt uncomfortable about doing so because of the lack of empathetic care and they feared undue criticism by health professionals [18].

### A matter of teamwork: recommendations for improvement

It is paramount that health professionals acknowledge the knowledge and hard work that women and their families put into managing diabetes in order to avoid frustrations and disappointments. Discussing and agreeing upon realistic and achievable diabetes outcomes will enhance mutual trust and confidence in the relationship. In addition, there is a great need for professionals to recognise the positive experience of pregnancy and focus more on maximising the expectant mother’s experience.

An Australian study found that women with T1DM considered it important to involve family members in their diabetes management during transition to motherhood, particularly during pregnancy and early motherhood, when the women felt torn between their babies’ needs and the requirement of their diabetes regimens [14]. Managing motherhood and diabetes is a balancing act [40] and increases women’s dependence on their partners and their own mothers [19]. Health professionals need to be sensitive to the powerful influences that their values and attitudes may have on the self-management decisions made by women with T1DM [41].

Health professionals can help by paying attention to attributes of expertise in everyday diabetes management [19,41,42], in particular, how women assess risks, compare current with previous experiences, seek explanations for changing BGLs, choose actions, and evaluate their decisions [43]. It is critical to successful transitions that, simultaneous to forming meaningful relationships, women have to take control of their fluctuating BGLs to avoid unpredictable events such as hypo- or hyperglycaemia. Unpredictable experiences called forth unknown and unused resources essential for generating positive ways of responding and adapting to new situations [12].

Responding to new situations in positive ways depends on the resources under direct individual control and the resources accessible from family, friends, or the community [44,45]. Manageability largely depends on people experiencing a practical and physical sense of self-empowerment in coping with their biology and threats to their health [46]. The ability to reframe problems from a positive perspective requires ability to transform [47] and integrate [48] oneself in relation to diabetes management.

It is paramount that women with T1DM have access to updated and evidence-based information in order to make good decisions and achieve positive experiences in their transition to motherhood. This review found that such information was not always available to women, in particular those living in rural communities [28]. A study investigating opinions about care during pregnancy, childbirth and postnatally among women with T1DM and gestational diabetes found that staff in different areas of care might need more knowledge about diabetes to reduce the risk of women receiving inadequate/incorrect information [49]. The authors concluded more effort must be expended on procedures and written materials, to ensure that women are better prepared and that the information flow throughout the care chain is reliable.

### Need for evidence-based information and development of web-based support

Internet use and web-based information plays an important role in delivering information to pregnant women with T1DM [34]. A high proportion of women with T1DM seek diabetes-related information on the internet, especially before, during, and after pregnancy. An Australian study found that websites and e-mails had a positive impact on the self-perceptions, self-confidence and diabetes management of young women with T1DM by making it easier to access information [6]. A UK study found that women sought formal sources of biomedical information from trusted sites, such as medical organisations and journals, and informal sources of personal stories to validate their own emotional journeys. Consequently, their usage of the internet helped women with problematic pregnancies feel more ‘normal’ [50]. In addition, internet use reduced their sense of isolation and informed them about different approaches to health services [6]. These findings highlight the importance of further developing effective web-based support that contains reliable information, interactive support and enables social networking for this population [34].

### Limitations of the review

Many of the studies had small samples sizes and inappropriate control groups. Despite the small numbers of women with T1DM, it is clear that these issues are vital for health professionals to address when assisting women with T1DM to achieve optimal well-being in their transition to motherhood. The review included only articles published in English. The synthesis was conducted on the basis of the findings and results published in the selected articles and limited to the chosen databases. In addition, the review represents the authors’ interpretation of other researchers’ interpretations of their data. Most quantitative studies used general measures of anxiety, depression and distress rather than diabetes-specific measures, which may make it difficult to apply the findings to the diabetes population.

### Future research

This review was part of a program of research that led to the development of two questionnaires (a pregnancy and a postnatal version) specifically assessing the psychosocial needs of women with T1DM transitioning to motherhood [51]. To our knowledge, this is the first such questionnaire to incorporate items covering both diabetes and pregnancy, and focusing on psychological well-being, concerns relating too physical well-being (maternal and fetal) and the social environment of the woman. We expect that these new measures will enable health professionals and researchers to better identify women’s support needs during pregnancy and in the immediate postnatal period.

Although this review specifically focussed on women with T1DM, there is also a need for further research comparing pregnant women with T1DM or T2DM and women with gestational diabetes in respect to their emotional well-being. Another area of research that needs attention is the partner’s and families’ experiences during the different stages of pregnancy and early motherhood.

## Conclusion

This structured literature review of 16 empirical studies revealed that decision-making about sharing responsibility for diabetes management among pregnant women, their immediate social network and health professionals is integral to positive transitional experiences for women with T1DM. The supportive role of partners was particularly important to the women during all phases of the transition. It is paramount that health professionals recognise the changing needs of women through the different stages of pregnancy. The review raised several questions and we recommend further research using instruments specifically designed for this group. Further research is needed to determine the psychosocial impact and associated behavioural outcomes of women with T1DM during the different transition phases to motherhood. A better understanding of the psychosocial issues will be instrumental for the design of interventions to support pregnant women and new mothers living with T1DM.

### Additional material

The Process of the of selecting studies for the review (Figure 1).

Summary of sample (Table 1).

Methodological details of included studies (Additional file 1).

## Abbreviations

T1DM: Type 1 diabetes mellitus; BGLs: Blood glucose levels; HbA1c: Glycosylated haemoglobin.

## Competing interests

The authors declare that they have no competing interests.

## Authors’ contributions

BR collected data, performed the review and BR drafted the manuscript. BC assisted in performing the review and commented on drafts, TD provided advice throughout the study, commented and assisted in editing the manuscript, CH collected data, assisted in drafting manuscript and provided substantial advice throughout the study, MB assisted in conceptualising the study and provided advice, JS provided substantial advice on the design, provided advice throughout the study and commented and edited the manuscript. All authors have approved the final manuscript.

## Pre-publication history

The pre-publication history for this paper can be accessed here:

http://www.biomedcentral.com/1471-2393/13/218/prepub

## Supplementary Material

Additional file 1: Table S1 Methodological details of the included studies on women with T1DM experience of transition to motherhood. Click here for file

## References

[B1] Australian Institute of Health and Welfare How Common is Diabetes 2011 AIHW http://www.aihw.gov.au/how-common-is-diabetes/

[B2] Australian Institute of Health and Welfare Diabetes in Pregnancy: Its Impact on Australian Women and their Babies 2010 Canberra: AIHW 2010; Diabetes series no.14 Cat.no.CVD 52

[B3] Kinsley B Achieving better outcomes in pregnancies complicated by type 1 and type 2 diabetes mellitus Midwifery 2007 29 S153 S160 10.1016/j.clinthera.2007.12.015 18191067

[B4] Masson EA Patmore JE Brash PD Baxtert M Caldwell G Gallens G Price PA Vice PA Walkert JD Lindow SW Pregnancy outcome in Type 1 diabetes mellitus treated with insulin lispro (Humalog) Diabetes Med 2003 20 46 50 10.1046/j.1464-5491.2003.00840.x 12519319

[B5] Nielsen LR Pedersen-Bjergaard U Thorsteinsson B Johansen M Damm P Mathiesen ER Hypoglycemia in pregnant women with type 1diabetes: predictors and role of metabolic control Diabetes Care 2008 31 9 15 17909091 10.2337/dc07-1066

[B6] Rasmussen B Dunning T O’Connell B Young women with diabetes: using Internet communication to create stability during life transitions J Clin Nurs 2007 16 17 24 10.1111/j.1365-2702.2006.01657.x 17518865

[B7] Bridge W Transitions: Making Sense of Life’s Changes 2001 London: Positive Paperbacks, Nicholas Brealey Publishing

[B8] Bridge W Managing Transitions: Making the Most of Change, People Skills for Professionals 2002 London: Positive Paperbacks, Nicholas Brealey Publishing

[B9] Chick N Meleis AI Transitions: A Nursing Concern. In Nursing Research Methodology: Issues and Implementation 1986 Maryland, Rockville: Aspen Publishers 237 257

[B10] Wheaton B Life transitions, role histories, and mental health Am Sociol Rev 1990 55 209 223 10.2307/2095627

[B11] Antonovsky A Health, Stress, and Coping 1979 San Francisco: Jossey-Bass Publisher

[B12] Antonovsky A Unravelling the Mystery of Health: How People Manage to Stay Well 1987 San Francisco: Jossey-Bass Publisher

[B13] Arnett JJ Young people’s conceptions of the transition to adulthood Youth Soc 1997 29 3 14 10.1177/0044118X97029001001

[B14] Rasmussen B O’Connell B Dunning P Cox H Young women with type 1 diabetes management of turning points and transitions Qual Health Res 2007 17 300 310 10.1177/1049732306298631 17301339

[B15] Anderberson BJ Families and chronic illness research: targeting transitions and tools– commentary on Trief et al Fam Syst Health 2006 24 332 335

[B16] Ringholm L Pedersen-Bjergaard U Thorsteinsson B Damm P Mathiesen ER Hypoglycaemia during pregnancy in women with Type 1 diabetes Diabetic Med 2012 29 5 558 566 10.1111/j.1464-5491.2012.03604.x 22313112

[B17] Rasmussen B Ward G Jenkins A King SJ Dunning T Young adults’ management of Type 1 diabetes during life transitions J Clin Nurs 2011 20 1981 1992 10.1111/j.1365-2702.2010.03657.x 21545569

[B18] Stenhouse E Letherby G Mother/daughter relationships during pregnancy and the transition to motherhood of women with pre-existing diabetes: raising some issues Midwifery 2011 27 2 120 124 10.1016/j.midw.2009.06.004 19640622

[B19] Stenhouse E Letherby G Stephen N Women with pre-existing diabetes and their experiences of maternity care services Midwifery 2012 doi:10.1016/j.midw.2011.12.007 10.1016/j.midw.2011.12.007 22721838

[B20] Gupton A Heaman M Cheung LW Complicated and uncomplicated pregnancies: women’s perception of risk JOGNN 2001 30 192 201 10.1111/j.1552-6909.2001.tb01535.x 11308109

[B21] Heaman M Gupton A Gregory D Factors influencing pregnant women’s perceptions of risk MCN Am J Matern Child Nurs 2004 29 111 116 10.1097/00005721-200403000-00010 15028919

[B22] Sparud-Lundin C Marie B Extraordinary exposed in early motherhood-a qualitative study exploring experiences of mothers with type 1 diabetes’ BMC Womens Health 2011 11 1 9 10.1186/1472-6874-11-1 21473755 PMC3079679

[B23] Lavender T Platt MJ Tsekiri E Casson I Byrom S Baker L Walkinshaw S Women’s perceptions of being pregnant and having pregestational diabetes Midwifery 2010 26 589 595 10.1016/j.midw.2009.01.003 19250724

[B24] Burns N Grove SK The Practice of Nursing Research: Appraisal, Synthesis, and Generation of Evidence 2009 6 Philadelphia: Saunders/Elsevier Publishing 104 111

[B25] Berg M Honkasalo ML Pregnancy and diabetes: a hermeneutic phenomenological study of women’s experiences J Psychosom Obst Gyn 2000 21 39 48 10.3109/01674820009075607 10907214

[B26] Berg M Pregnancy and diabetes: how women handle the challenges J Perinat Educ 2005 14 23 32 10.1624/105812405X57552 17273439 PMC1595250

[B27] Berg M Sparud-Lundin C Experiences of professional support during pregnancy and childbirth - a qualitative study of women with type 1 diabetes BMC Pregnancy Childbirth 2009 9 27 34 10.1186/1471-2393-9-27 19575789 PMC2725032

[B28] King R Wellard S Juggling type 1 diabetes and pregnancy in rural Australia Midwifery 2009 25 126 133 10.1016/j.midw.2007.01.016 17509737

[B29] Dalfra M Nicolucci A Bisson T Bonsembiante B Lapolla A Quality of life in pregnancy and post-partum: a study in diabetic patients Qual Life Res 2011 15 238 242 10.1007/s11136-011-9940-5 21633879

[B30] Illias I Papageorgiou C Katsadoros K Zapanti E Anastasiou E Preliminary report: psychological assessment of Greek women with diabetes during pregnancy Percept Motor Skill 2005 101 3 10.2466/pms.101.2.628-630 16383101

[B31] Langer N Langer O Pre-existing diabetics: relationship between glycemic control and emotional status in pregnancy J Matern Fetal Med 1998 7 257 263 9848689 10.1002/(SICI)1520-6661(199811/12)7:6<257::AID-MFM1>3.0.CO;2-H

[B32] Levy-Shiff R Lerman M Har-Even D Hod M Maternal adjustment and infant outcome in medically defined high-risk pregnancy Dev Psychol 2002 38 93 103 11806705

[B33] Moore ML Meis P Jeffries S Ernest JM Buerkle L Swan M Hill CA A comparison of emotional state and support in women at high and low risk for preterm birth, with diabetes in pregnancy, and in non-pregnant professional women J Prenat Perinat Psychol Health 1991 6 2 109 127

[B34] Sparud-Lundin C Ranerup A Berg M Internet use, needs and expectations of web-based information and communication in childbearing women with type 1 diabetes BMC Med Inform Decis Mak 2011 11 49 55 10.1186/1472-6947-11-49 21736713 PMC3141376

[B35] Spirito A Ruggiero L Coustan D McGarvey S Bond A Mood state of women with diabetes during pregnancy J Reprod Infant Psych 1992 10 29 38 10.1080/02646839208403266

[B36] Ruggiero L Spirito A Coustan D McGarvey ST Low KG Self-reported compliance with diabetes self-management during pregnancy Int J Psychiat Med 1993 23 195 207 10.2190/6L8X-YJP0-4G3D-HUUJ 8360001

[B37] York R Brown LP Persily CA Jacobsen BS Affect in diabetic women during pregnancy and postpartum Nurs Res 1996 45 54 56 10.1097/00006199-199601000-00010 8570424

[B38] Langer N Langer O Comparison of pregnancy mood profiles in gestational diabetes and pre-existing diabetes Diabetes Educator 2000 26 4 667 672 10.1177/014572170002600414 11140075

[B39] Rasmussen B Turning Points and Transitions: Experiences of young women with Type1 diabetes. PhD Thesis 2005 Melbourne: Deakin University

[B40] Poirier-Solomon L A balancing act: managing motherhood and diabetes Diabetes Forecast 2002 55 46 49 14768603

[B41] Thorne SE Nyhlin KT Paterson BL Attitudes towards patient expertise in chronic illness Int J Nurs Stud 2000 37 303 311 10.1016/S0020-7489(00)00007-9 10760537

[B42] Paterson BL Thorne SE Developmental evolution of expertise in diabetes self-management Clin Nurs Res 2000 4 402 419 11881697 10.1177/10547730022158663

[B43] Paterson BL Thorne SE Expert decision making in relation to unanticipated blood glucose levels Res Nurs Health 2000 23 47 57 10.1002/(sici)1098-240x(200004)23:2<147::aid-nur7>3.0.co;2-s 10782873

[B44] Rayman KM Ellison GC Home alone-The experience of women with type 2 diabetes who are new to intensive control Health Care Women Int 2004 25 900 915 10.1080/07399330490508604 15513798

[B45] Schlossberg NK Waters EB Goodman J Counselling Adults in Transition: Linking Practice with Theory 1995 2 New York: Springer

[B46] Sanden-Erikson B Coping with type-2 diabetes: the role of sense of coherence compared with active management J Adv Nurs 2000 31 1393 1397 10.1046/j.1365-2648.2000.01410.x 10849151

[B47] Paterson BL The shifting perspectives model of chronic illness J Nurs Scholarship 2001 33 21 26 10.1111/j.1547-5069.2001.00021.x 11253576

[B48] Hernandez CA Integration: the experience of living with insulin-dependent diabetes Can J Nurs Res 1996 28 37 56 9128475

[B49] Anderberg E Berntorp K Crang-Svalenius E Diabetes and pregnancy: womens’ opinions about the care provided during the childbearing year Scand J Caring SCI 2009 23 161 170 10.1111/j.1471-6712.2008.00614.x 19192239

[B50] Lowe P Powell J Griffiths F Thorogood M Locock L “Making it All normal”: the role of the internet in problematic pregnancy Qual Health Res 2009 19 1476 1484 10.1177/1049732309348368 19805809

[B51] Rasmussen B Dunning T Hendrieckx C Botti M Speight J Transition to motherhood in type 1 diabetes: design of the pregnancy and postnatal well-being in transition questionnaires BMC Pregnancy Childbirth 2013 13 54 10.1186/1471-2393-13-54 23445534 PMC3599343

