# Supplementary material for: Psychosocial issues of women with type 1 diabetes transitioning to motherhood: a structured literature review
Source: BMC Pregnancy Childbirth. 2013 Nov 23;13:218. doi: 10.1186/1471-2393-13-218 (PMC4222685; doi:10.1186/1471-2393-13-218)
Supplement: Additional file 1: Table S1 — Methodological details of the included studies on women with T1DM experience of transition to motherhood. [file 1471-2393-13-218-S1.docx]

**Table 2: Methodological details of the included studies on women with T1DM experience of transition to motherhood**

| **Authors**  **Year**  **Country** | **Sample (n)**  **Age years (SD or range)**  **Ethnicity** | **Article** | **Study Design** | **Key aims**  **Method**  **Outcome measures** | **Key findings** |
| --- | --- | --- | --- | --- | --- |
| Berg M and Honkasalo ML  2000  Sweden | N=14 pregnant women with type 1 diabetes  28 (25-38)  NR | Pregnancy and diabetes - a hermeneutic phenomenological study of women’s experiences ([1](#_ENREF_1)) | Hermeneutic phenomenological reflective life world approach. | To describe the ‘lifeworld’ of the everyday experience of women with type 1 diabetes during pregnancy; in particular the crucial elements of the pregnancy experience and whether the experience varies throughout the pregnancy.  Women described their experience at different stages during pregnancy (37 interviews) and one week post partum (7 interviews) | Two themes were identified   1. objectification: the ‘unwell body at risk’, this was a similar experience to when first diagnosed with diabetes; loss of control, ‘wishing for a normal life with control’, ‘not knowing their own body’, as control decreased, confidence was lost and frustration increased. 2. exaggerated responsibility; there was a ‘constant worry’, women felt ‘constantly under pressure’, and were ‘constantly self-blamed’ when anything went wrong, such as experiencing complications; the women’s moral commitment towards the new growing child,   Themes did not differ between phases. |
| Berg M  2005  Sweden | N=18 pregnant women with type 1 diabetes  4 interviews added to update data.  28 (25-38)  NR | Pregnancy and diabetes: How women handle the challenges([2](#_ENREF_2)) | Hermeneutic phenomenological reflective life world approach. | To determine how women with type 1 diabetes handled their daily life during pregnancy.  Women described their experience at different stages during pregnancy (41 interviews) and one week post partum (7 interviews) | The ‘essential’ structure of the phenomenon which was summarised as “to master or to be enslaved”’, included three major constituents:   1. meaningfulness/ meaninglessness, 2. reconciliation/ conflict, 3. shared control/ unwillingly controlled. |
| Berg M and  Sparud-Lundin C  2009  Sweden | N=23 women with type 1 diabetes, 6-24 months after delivery  32 (22-37)  NR | Experiences of professional support during pregnancy and childbirth – a qualitative study of women with type 1 diabetes (3) | Hermeneutic phenomenological reflective life world approach. | To explore need for and experience of professional support during pregnancy and childbirth among women with type 1 diabetes.  Six focus groups including a total of 19 women and four individual interviews were conducted. The participants were encouraged to narrate their experiences of pregnancy and childbirth in relation to glycaemic control, well-being and provided care. | Five themes of meaning were identified during pregnancy:   1. The women felt under pressure and worried/guilty about jeopardizing the baby's health. 2. This was sometimes made worse by care providers' manner and lack of competence and support. 3. The increased attention from care providers during pregnancy was experienced as related to the health of the unborn child; not the mothers. 4. Women who during pregnancy received care in a disconnected diabetes organisation were forced to act as messengers between different care providers. 5. Need to share experiences with other pregnant women. 6. Feeling abandoned by midwives and left with responsibility for BGLs during birth, which triggered anxiety and uncertainty. 7. Need to stay in control of BGLs to have a sense of security 8. Both trust and distrust in providers’ specific diabetes-obstetric competence. 9. A need to clarify responsibility for BGL control both among care providers and the women and care providers. |
| Dalfrà MG,  Nicolucci A,  Bisson T,  Bonsembiante B,  Lapolla A  2011  Italy | N= 245 pregnant women  30 T1DM  176 GDM  39 women without diabetes  T1DM: 31.7 (3.8)  GDM: 33.9 (4.5)  Controls: 31.8 (4.4)  Caucasian | Quality of life in pregnancy and post-partum: a study in diabetic patients (4) | Prospective cohort study | To evaluate quality of life in pregnant women with diabetes followed up at diabetes clinics.  In third trimester of pregnancy and 8 weeks after delivery participants completed:   - SF-36 Health Survey - Center for Epidemiological Studies-Depression (CES-D) Scale   GDM and T1DM completed two diabetes-specific scales on diabetes-related stress and diabetes health distress | In the third trimester of pregnancy:  - women with T1DM or GDM had a better Standardised Physical Component score than control group (P<0.0001, P = 0.009, resp).  - women with T1DM or GDM scored significantly lower for general health perception than control group (P<0.0001, P = 0.009, resp)  - women with T1DM had lower Standardised Mental Component scores than control group (P = 0.03).  No differences between groups for depression and diabetes-related stress or distress.  Compared to third trimester of pregnancy:  - severity of depressive symptoms increased after delivery in both diabetic groups, and dropped in controls (P=0.0001).  - scores improved in all SF-36 areas in healthy controls and women with GDM, while they all became worse in the T1DM group. |
| **Ilias J, Papageorgiou C**  **Katsadoros K, Zapanti E, Anastasiou E**  **2005**  **Greece** | N=36  23 T1DM  13 GDM  TD1M 29.5 (4.1)  GDM 28.9 (3.5)  Greek | Preliminary report : Psychological assessment of Greek women with diabetes during pregnancy ([3](#_ENREF_3)) | Cross-sectional prospective cohort study | Aim of study not recorded  Women completed validated Greek versions of questionnaires in the second and third trimester of pregnancy:   - Maudsley Obsessive-Compulsive Inventory - Toronto, alexithymia scale - Zung Depression scale - Spielberger State-Trait Anxiety Inventory (STAIT) | Compared to women with GDM women with T1DM   - had a higher mean score for obsessive-compulsiveness in 2^nd^ and 3^rd^ trimester of pregnancy - had higher mean scores for alexithymia, depression and anxiety in the 2^nd^ , but not in the 3^rd^ trimester   . |
| King R and Wellard S  2009  Australia | N=7 women T1DM, who had given birth within past 12 months  30.24 (26-35)  NR | Juggling type 1 diabetes and pregnancy in rural Australia ([4](#_ENREF_4)) | Case-study design | To explore the experiences of women with type 1 diabetes, living in rural Australia, while preparing for pregnancy and childbirth.  To describe women’s engagement with, and expectations of, health-care providers during this period, and subsequently highlight potential service and informational gaps.  In-depth face-to-face or telephone interviews were conducted over a 3 month period. Descriptions of preparation and experiences of child birth and healthcare during the perinatal period were the focus. | Women with diabetes recognised than pregnancy posed a greater strain upon them compared to pregnant women without diabetes. Pregnancy was dominated by glucose management and its consequence effect (e.g. loss of recognition and change of symptoms of hypos, and higher frequency of hypos). Discipline and hard work was required for blood glucose management.  Women reported difficulties in obtaining healthcare provider/services support and appropriate information. |
| Langer N and Langer O  1998 and 2000  USA  *(two publications same study)* | Second analysis of study 1999  N=100 PGDM  N=206 GDM,  N=132 controls  PEDM  80% Mex-Amer.  14% non-Hisp Amer.  6% African-Amer.    Controls  78% Mex-Amer.  14% non-Hispanic American  8% African-Amer.  PGDM and control group: see previous study  GDM: not reported  NR | Comparison of pregnancy mood profiles in gestational diabetes and pre-existing diabetes ([5](#_ENREF_5)) | Cross sectional prospective cohort study | To identify the impact of intensified diabetes management on emotional profiles and relationship between glycemic control and emotional profiles.  Women completed the Profile of Mood States-Bipolar (POMS-BI) questionnaire at 37 to 38 weeks of pregnancy. | *Type of PEDM not specified.*  For women with PGDM there was no association between level of glucose control and mood disturbances.  PGDM group displayed greater depression compared with GDM and control groups (P<0.004; P<0.02 resp.).  PGDM group reported significantly greater levels of hostility compared to GDM group (P<0.01) |
| Lavender T,  Platt MJ,  Tsekiri E,  Casson I,  Byrom S,  Baker L,  Walkinshaw S.  2010  UK | N= 22 women T1DM 15 (10 pregnant)  NR  Range 20- 42  14 Caucasian  7 South East Asian Ethnicity  1 African ethnicity | Women's perceptions of being pregnant and having pregestational diabetes | A hermeneutic phenomenological approach | To explore the experiences of White British and South East Asian women with type 1 and type 2 diabetes, and the perceived impact of diabetes on their reproductive health.  Focus groups and one-to-one interviews were used to elicit women’s experiences. | Three main themes were identified: relinquishing personal control, pregnancy overshadowed by diabetes and haphazard preconception care.  Key conclusions: strategies should be developed to ensure that whilst safety is maintained, the pregnancy focus is not lost. Women should be supported to optimise their experience as well as clinical outcomes. The convergence of professional roles needs consideration; individual members of multidisciplinary diabetes teams should provide a unique and complementary contribution to care. Preconception care needs to be accessible and responsive to women; this should include recognition of socio-cultural differences. |
| Levy-Shiff R, Lerman M,  Har-Even D,  Hod M  2002  Israel | N=153 pregnant women  53 PGDM (insulin treated)  51 GDM  49 non-diabetic  PGDM: 31.5 (5)  GDM: 32.6(4.9)  Control: 31.8 (5)  Israeli women | Maternal adjustment and infant outcome in medically defined high-risk pregnancy | Cross-sectional prospective cohort study | To explore the association between diabetic pregnancy and women’s psychosocial functioning by comparing PGDM women, GDM women, and nondiabetic women undergoing low-risk pregnancies and  the relative contributions of the pregnancy’s medical status and of maternal coping and resources in predicting pregnancy outcome.  Psychosocial processes, using the stress-and-coping model.  Questionnaires were completed in the second trimester   - Cognitive Appraisal of Pregnancy - Cognitive Appraisal of Control - Ways of Coping Checklist - Social Support Questionnaire, Pregnancy-Related Emotions - Beck Depression Inventory-R - State trait Anxiety Inventory - Burnout questionnaire - Symptom Checklist | During pregnancy, women with PGDM and GDM raised more intense pregnancy-related negative emotions (e.g. disappointment, fear, quilt, worry) and fewer positive emotions (e.g. confidence, hope, eagerness, exhilaration) compared to control group (P<.01). However, their overall well-being (e.g. depression, anxiety) was not found to be adversely impacted by their higher-risk.  Control of diabetes also significantly correlated with social support satisfaction.  In the PGDM group, higher levels of medical support were significantly associated with lower levels of anxiety and depression (P <.01), and activity-focused strategies were found to be negatively related to anxiety and negative emotions (P <.01); such associations were insignificant in the control group. |
| Moore ML,  Meis P,  Jeffries S,  Ernest JM,  Buerkle L,  Swain M  Hill C.  1991  USA | N=131  T1DM = 73  High Risk = 48  Low Risk = 25  Additional professional women not pregnant = 16  Women with T1DM as comparison group  NR  Caucasian = 77 | A comparison of emotional state and support in women at high and low risk for preterm birth, with diabetes in pregnancy, and in non-pregnant professional women ([6](#_ENREF_6)) | Cross-sectional descriptive study | To examine psychosocial factors involved in producing pregnancy complications.  Women completed seven instruments between 24 and 30 weeks gestation:   - three measures of ‘distress’ (STAIT –State; Depression Adjective Checklist, Perceived Stress Scale) - two measures of support (Perceived Social Support -Friends scale & Family scale) - a measure of life event - Eysenck Personality Inventory (Neuroticism) | Women of Caucasian ethnicity, with diabetes who attended a private clinic had the highest mean scores on all distress measures compared to high and low risk groups, and professional groups (P value not reported).  Women with diabetes attending public clinics reported the lowest level of friends support compared to other groups.  When grouped by age (≤19 or >19 yrs), in the young age group a pattern of increased distress, undesirable and total life events and lower friends support was found in adolescents with diabetes and high risk preterm women.  Overall the results suggest that economic status is as important as medical risk as a source of distress among pregnant women. Assessment of life events and support in prenatal stage seemed to be important. |
| Ruggiero L,  Spirito A,  Coustan D.  McGarvey ST,  Low KG  1993  USA | N=49 pregnant women with  type 1 (68%)  type 2 (32%) diabetes  26.75 (5.1)  92% Caucasian | Self-reported compliance with diabetes self-management during pregnancy | Longitudinal study | To examine regimen compliance in pregnant women with pre-existing diabetes across multiple self-care tasks at three times during the pregnancy: mid-second, early third and late third trimesters  Women completed following measures at mid second, early and late third trimester:   - Diabetes Compliance Q - Diabetes Social Support Q - The Hassles Scale | *Because no differences were found between women with T1DM or T2DM on any of the compliance scales, analyses performed for total sample*.  Women reported being highly compliant for insulin administration (86-88%), in managing insulin reactions (85-89 %), and for glucose testing ( 94-96%), but less for diet (74-79%). (No sign differences across pregnancy)  Major and minor life events and regimen-related social support were significantly related to self-reported diet compliance. |
| Sparud-Lundin C,  Berg M  2011  Sweden | N=23 T1DM  (see Berg and Sparud-Lundin, 2009)  NR (range 22 and 37 yrs)  Swedish born | Extraordinary exposed in early motherhood – a qualitative study exploring experiences of mother with type 1 diabetes (11) | Hermeneutic phenomenological reflective life world approach. | To explore experiences after childbirth regarding breastfeeding, glycemic control, support  and well-being in women with type 1 diabetes  Focus groups and interviews | After childbirth the mothers felt abnormal in comparison to other mothers mainly because of extra care requirements. Transition into motherhood was challenging and included a struggle with breastfeeding, although with a driving force to succeed.  Everyday life was filled with uncertainty and unpredictability related to one’s own unstable glycemic control and the women down-prioritized their own needs in favour of the child. A feeling of being disconnected from professional care further contributed to challenges. |
| Sparud-Lundin C  Ranerup A,  Berg M  2011  Sweden | N=105 mothers with T1DM and recent childbearing experience.  ≤30: 28 women 31-35: 40  >36: 37 | Internet use, needs and expectations of web-based information and communication in childbearing women with type 1 diabetes | Web-based survey with explorative and descriptive design | To explore internet use, needs, and expectations regarding web-based information and communication in childbearing women with type 1 diabetes.  Web-based survey specifically designed for this project | Of the 105 women, 12% searched for information every day, 29% one or more times a week, and 38% one or more times a month, 22% never used the internet to search for information concerning pregnancy, childbirth, and parenthood. 45 % said to be active participants. 45% of the women expressed a (quite) great need for web-based support related to pregnancy, childbirth and parenthood, especially those with higher educational level (P = .01). Main finding:   - Expectations of instrumental and informational support included an expert-controlled website with reliable, - updated, and information focused on childbearing and diabetes, improved access to diabetes care professionals and alternative ways to communicate and to receive childbearing-related support. - Online technical devices to manage the frequent monitoring of blood glucose during pregnancy. - Informal, emotional, and appraisal support from women in similar situations was suggested |
| Spirito a, Ruggieor L, Coustan D, McGarvey S, Bond A  1992  US | N=106 pregnant women  43 PEDM  63 control  PEDM: 25.5 (4.5)  Control 27.3 (4.5)  PEDM: 90.7% Caucasian  Control: 86.8% Caucasian | Mood state of women with diabetes during pregnancy | Not recorded | To examine the stability of mood state in pregnant women with diabetes and to compare their functioning to nondiabetic pregnant women.  Semi-structured interviews at second and third trimester of pregnancy including Profile of Mood States-bipolar Form (POMS)  Two scales excluded (confounders with being pregnant and diabetes): tired-energetic/clearheaded-confused | *Type of PEDM not specified*.  Mean scores for both groups fell within normal range.  Women with PEDM were more anxious (P<0.05) and tended to be more depressed than women without diabetes. The mood state remained stable between the two assessments.  The percentage of women with PEDM with clinically significant levels of anxiety was higher than control group on anxiety (3^rd^ trimester, 25.6 vs 8.8%; p<0.05). |
| Stenhouse, E,  Letherby, G  Stephen, O  2012  UK | N=20  8 T1DM  4 T2DM  1 group including mother and father  3 including mothers  2 including partners  Ethnicity NR | Women with pre-existing diabetes and their experiences of maternity care services | Grounded theory using in-depth qualitative interviews with 6 interviews in dyad with partners, mothers and father. | To explore the experience of maternity care services used by women whose pregnancy is complicated by pre-existing diabetes, to gain a deeper under- standing of service use and to identify aspects of services that women with pre-existing diabetes would like improved.  A convenience sampling procedure was used. Respondents consisted of 12 pregnant women with T1DM and T2DM and eight of their significant others. | Three themes were identified from interviews: empathic care with care more focused on diabetes not pregnancy; feeling judged by health-care professionals (with nearly all respondents reporting negative encounters of consultation with the specialist team); and the notion of expertise (with respondents reporting feeling frustrated when it seemed health-care professionals did not value their expertise). |
| York R,  Brown LP,  Persily CA, Jacobsen BS  1996  USA | N=36  6 PGDM,  30 GDM  27.8 (5.5)  81% African-American,  19% Caucasian | Affect in diabetic women during pregnancy and postpartum (13) | Prospective cohort study | To provide baseline data on anxiety, depression, and hostility scores in women with diabetes in late pregnancy through 8 weeks postpartum  Women completed the Multiple Adjective Check List (MAACL) at five time intervals; 36 weeks gestation, 2 days postpartum, 1, 4 and 8 weeks postpartum. | *Type of PEDM not specified*.  During pregnancy, GDM women had higher anxiety (P=.012), and hostility (P=0.03) scores than women with PGDM diabetes, but did not have higher depression scores. No differences were observed in the postpartum period. |

BGLs: Blood Glucose levels; GDM: Gestational diabetes mellitus ; PEDM: Pre-existing diabetes mellitus; PGDM: Pregestational diabetes mellitus; T1DM: Type 1 diabetes mellitus; T2DM: type 2 diabetes mellitus; NR: Not recorded
